# A Bronchoscope Localization Method Using an Augmented Reality Co-Display of Real Bronchoscopy Images with a Virtual 3D Bronchial Tree Model

**DOI:** 10.3390/s20236997

**Published:** 2020-12-07

**Authors:** Jong-Chih Chien, Jiann-Der Lee, Ellen Su, Shih-Hong Li

**Affiliations:** 1 School of Informatics, Kainan University, Taoyuan 33857, Taiwan; jcchien@mail.knu.edu.tw; 2 Department of Electrical Engineering, Chang Gung University, Taoyuan 33302, Taiwan; ilasu0513@gmail.com; 3 Department of Neurosurgery, Chang Gung Memorial Hospital at Linkou, Taoyuan 33305, Taiwan; 4 Department of Electrical Engineering, Ming Chi University of Technology, New Taipei City 24301, Taiwan; 5 Department of Thoracic Medicine, Chang Gung Memorial Hospital at LinKou, Taoyuan 33305, Taiwan; penbus504@gmail.com

**Keywords:** augmented reality, bronchoscopy, localization

## Abstract

In recent years, Image-Guide Navigation Systems (IGNS) have become an important tool for various surgical operations. In the preparations for planning a surgical path, verifying the location of a lesion, etc., it is an essential tool; in operations such as bronchoscopy, which is the procedure for the inspection and retrieval of diagnostic samples for lung-related surgeries, it is even more so. The IGNS for bronchoscopy uses 2D-based images from a flexible bronchoscope to navigate through the bronchial airways in order to reach the targeted location. In this procedure, the accurate localization of the scope becomes very important, because incorrect information could potentially cause a surgeon to mistakenly direct the scope down the wrong passage. It would be a great aid for the surgeon to be able to visualize the bronchoscope images alongside the current location of the bronchoscope. For this purpose, in this paper, we propose a novel registration method to match real bronchoscopy images with virtual bronchoscope images from a 3D bronchial tree model built using computed tomography (CT) image stacks in order to obtain the current 3D position of the bronchoscope in the airways. This method is a combination of a novel position-tracking method using the current frames from the bronchoscope and the verification of the position of the real bronchoscope image against an image extracted from the 3D model using an adaptive-network-based fuzzy inference system (ANFIS)-based image matching method. Experimental results show that the proposed method performs better than the other methods used in the comparison.

## 1. Introduction

A lung biopsy operation under local anesthesia is used for the investigation of diffuse pulmonary lesions. The endobronchial navigation system is an important tool used in biopsy operations of the lung. This system depends on the images obtained during the surgery for the purpose of navigation and biopsy and places the burden for this on the surgeons. However, the approach is not without risks and visual interference factors. These factors include reflection caused the endoscope light source and bubbles generated by the saliva; i.e., areas of high reflectance. Thus, possible errors causing the misguidance of the endoscope may occur and cause the doctor to guide the scope down the wrong air passage. In 1981, a study of 4595 bronchoscopy procedures showed that complications developed in 235 cases, of which 51 cases had major complications [1]. It would be helpful to the surgeon to view the actual location of the endoscope relative to all of the passages of the trachea during surgery when facing this type of problem. Thus, in order to overcome this problem, this paper proposes a method using a virtual bronchial passage 3D model and matches the real bronchoscopy images to 2D slices of the 3D model in order to aid the surgeon in the determination of the actual position of the bronchoscope. The idea of combining virtual reality with surgery has been discussed in the literature [2,3]; this paper proposes a novel method as a way to use virtual reality with complex endobronchial surgery.

Most studies, including those by Mori [4], Hong [5] and the articles in [6], have agreed that a virtual bronchoscope examination system could help doctors to examine lesions, plan surgical paths, communicate with the patients about the surgical procedures, etc. Thus, this paper proposes a novel registration method to overlay real bronchoscopy images on top of virtual 3D bronchial tree models in an augmented reality-like system and then track the bronchoscope while displaying its current position. However, tracking the bronchoscope does not offer an easy solution because it is a nonlinear filtering problem, and thus various researchers have proposed different solutions. For example, Nagao [7] used the Kalman estimator to increase tracking accuracy. Helferty [8] proposed a method based on 2D image matching, which greatly increased tracking accuracy. Merritt [9] and Deligianni [10] proposed the calculation of the parameters of the bidirectional reflectance distribution function (BRDF) in non-real time to obtain the textures of the 2D bronchoscope images in order to enhance the realism of 3D computed tomography (CT) data. Based on this idea, it is possible to calculate the feature points of real bronchoscopy images using real-time methods to help track the bronchoscope path using similarity measures. Deguchi [11] proposed the use of the minimum square error (MSE) and modified minimum square error (MoMSE) as similarity measures to help track the regions-of-interest of real bronchoscopy images against the images from virtual 3D bronchoscopy. Mori [12] proposed an improved MoMSE algorithm which showed better performance in tracking than the original MoMSE. There have been other developments in 3D tracking in bronchoscopy based on the Oriented FAST and rotated BRIEF–Simultaneous Localization and Mapping (ORB-SLAM) [13] approach. ORB-SLAM uses the ORB, a binary feature, as a kernel to perform SLAM, which is used to estimate the pose and orientation of the monocular camera using ORB features. ORB-SLAM was used the basis for the tracking of an endoscope, and the tracking information was used to construct a 3D model of the organ in [14]. Wang, et al. [15] also used ORB as the basis for an improved visual SLAM for bronchoscopy application, which also used tracking data to construct a 3D model and achieved an average RMSE of 3.02 mm. However, the ORB feature was shown to be a worse performer in terms of feature matching accuracy than the Speed Up of Robust Features (SURF) approach in [16], and our approach differs from SLAM in that we verify the estimated position and orientation of the camera in the tracking stage by comparing the current real bronchoscope image against the images captured at the same position of a 3D virtual trachea in the verification stage. Thus, the method investigated in this paper is based on the proposed feature and image matching approach.

However, in a feature-based approach, in order to obtain correct similarity measures, interference areas caused by reflected glare from endoscope light must be removed from consideration in order to reduce errors. In dealing with the interferences, the proposed method uses an ANFIS (adaptive-network-based fuzzy inference system)-based [17] system to locate areas of interference in order to eliminate them during the search for the current location of the bronchoscope. For the search, a novel feature—KAZE [18]—with optical flow is integrated into the system. The organization of this paper is as follows: the proposed method will be discussed in Section 2. The experimental setup and comparison results with other methods will be presented in Section 3, followed by the conclusions in Section 4.

## 2. Method

First, the CT images of the patient’s tracheal system were used to build a 3D virtual bronchial tree model, which we called “V”. First, position tracking was performed by extracting two consecutive frames from a real bronchoscopy surgery video, B, which was later replaced with the images obtained during a real operation. The KAZE features were calculated for both frames and matched in order to obtain the movement from the first frame to the second frame. At the same time, the optical flow of these two same frames were calculated, which required more computational resources than the KAZE-based matching; an explanation for this design is discussed below. An ANFIS arbitration system was designed to select which of these vectors should be used to calculate the change in the camera’s position, ΔQ, which was used to calculate the current position of the camera. This information was passed to the virtual model to extract the current virtual frame, which was used for comparison with the current image from the actual video. However, before the comparison, areas within the actual image that caused a false match, such as areas with too much brightness and interference blocks, were first removed before matching, and an ANFIS system was used to determine whether an area should be removed. Finally, a fuzzy ANFIS system decided whether the virtual image was a good match for the actual image. If this was true, then the current position of the camera was assumed to be found, and then its location was displayed on the virtual model of the tracheal system alongside the actual image. If the ANFIS system decided that they were not a good match, an alternative ΔQ vector was found and another virtual frame was then extracted and compared until the ANFIS system agreed that they were a good match.

**Figure 1:**
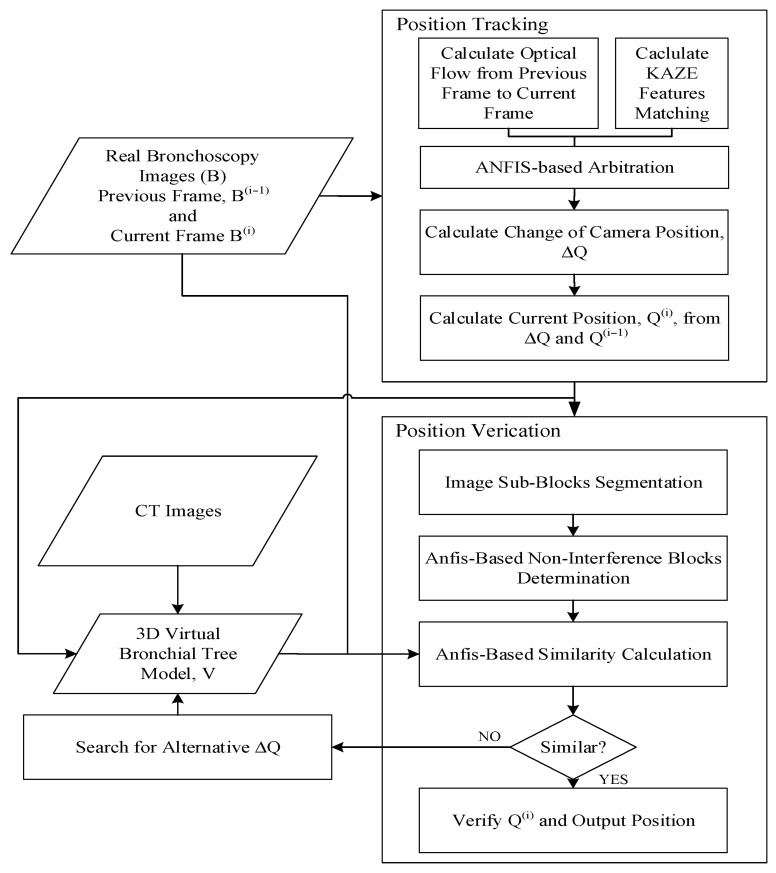
The system flowchart. ANFIS: adaptive-network-based fuzzy inference system.

The novelty of this system is that the localization is separated into two phases: tracking and verification. This is done so that the surgeon can quickly determine the current location of the bronchoscope in the virtual model of the trachea while viewing the output from the scope itself, even in the position tracking phase, and if necessary, the actual location will be corrected in the verification phase. The system flowchart of the proposed method is shown below in Figure 1.

### 2.1. Construction of 3D Bronchial Tree Model

**Figure 2:**
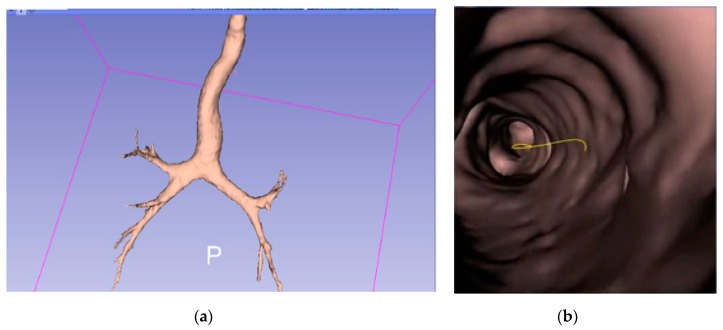
(**a**) Virtual 3D bronchial model constructed from computed tomography (CT) slices; (**b**) a sample virtual bronchoscopy image.

Given a set of CT Dicom (Digital Imaging and Communications in Medicine) images from the chest scan of a given patient, a 3D virtual bronchial model was built by connecting the point clouds representing the bronchial airways, and then the skin was pasted on, as shown below in Figure 2a. An example of the virtual bronchoscopy image is shown in Figure 2b.

**Figure 3:**
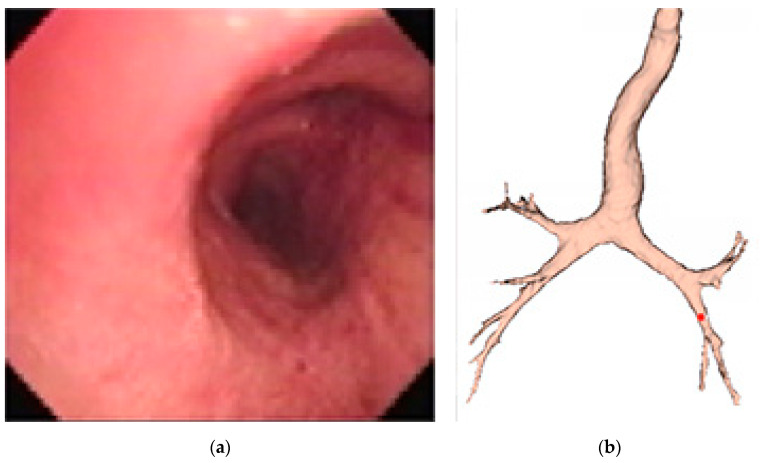
Simulation of augmented reality (AR) display of (**a**) the current real bronchoscopy image and (**b**) the virtual 3D model, with the location of bronchoscope displayed using a red dot.

The final expected resulting display is similar to that presented in Figure 3, where the red dot indicates the current location of the endoscope.

### 2.2. Position Tracking

In this paper, we propose the use of feature-based matching between consecutive frames from a bronchoscopy video in order to determine changes in position and orientation. To calculate the displacement, in this paper, we investigated three types of features. We compared SURF [19], Maximally Stable Extremal Regions (MSER) [20] and KAZE features. The SURF (Speed Up of Robust Features) approach, which uses square filters at different scales to perform image convolutions in a continuous manner to approximate Gaussian smoothing, then detects the immutable feature points within the integral image, S, which is defined as

(1) $$S\left(x,y\right)=\;\overset{x}{\underset{i=0}{\sum }}\overset{y}{\underset{j=0}{\sum }}I\left(i,j\right).\;$$

**Figure 4:**
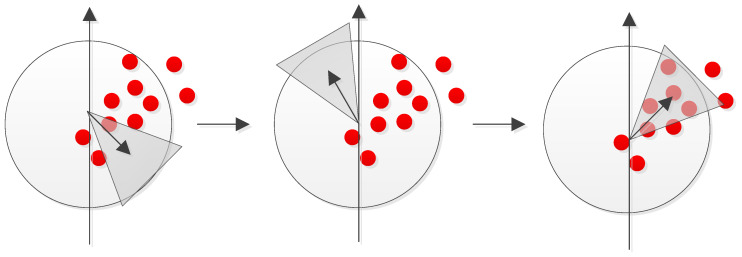
The search for the dominant orientation.

Because square filters are used, the integral images can be used to speed up the calculation. SURF uses a blob detector based on the Hessian matrix to detect the feature points by detecting the changes around the pixels of interest. In order to achieve rotational invariance, the direction of orientation of these pixels of interest must be found. Assuming that σ represents the scale at which a pixel of interest is found, and that the Haar wavelet [21] responses in both *x* and *y* directions have a radius of 6σ around the feature point, then the dominant orientation is determined by calculating the sum of all responses within a sliding window. The resultant descriptor for each feature point is a description with 64 dimensions. The following figure, Figure 4, shows the search for the dominant orientation.

The MSER (Maximally Stable Extremal Regions) is a technique that is known to able to find correspondences between elements from two images. It defines regions as contiguous subsets within an image, and extremal regions as those regions in which the intensities of all points within the regions are higher than the intensities of the points at the boundary of the region. Thus, MSER shows the extremal regions that are almost uniform in intensities and surrounded by contrasting backgrounds.

KAZE is a novel feature/method that operates in a nonlinear scale space, unlike SURF, which has partial accuracy ambiguity due to the use of linear scale space. It uses nonlinear diffusions and keeps important feature points in nonlinear-scale spaces. The method it uses to obtain the nonlinear scale space is AOS (additive operator splitting), which is based on the splitting of a complex problem into a sequence of similar tasks. The dominant orientation is found using a method similar to SURF.

A preliminary comparison was done between these three types of features. Although each type of feature may have different suitabilities in different circumstances, in this paper, we sought to identify the most efficient feature to be used for a bronchoscopy video. The images from the bronchoscopy video are usually of the tracheal tunnel in the middle, surrounded by the tracheal wall. The middle of the image is relatively clear and discernable, but the surrounding wall is blurred due to the camera being too close to the lens, and the image is also distorted due to the fisheye shape of the lens.

**Figure 5:**
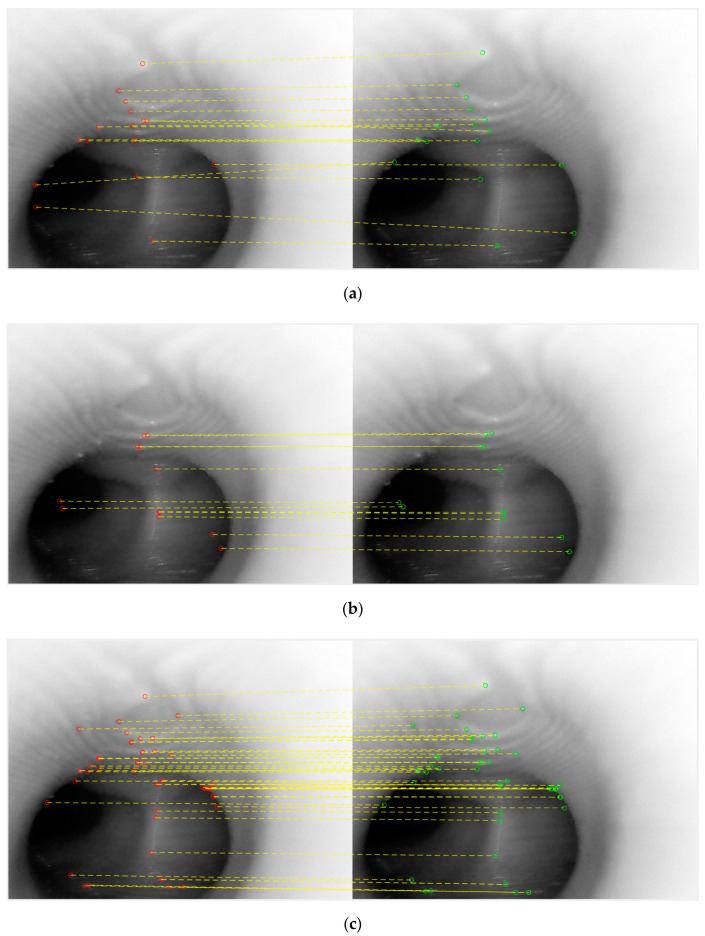
The matching results of the three methods: (**a**) Speed Up of Robust Features (SURF), (**b**) Maximally Stable Extremal Regions (MSER) and (**c**) KAZE.

We extracted two consecutive images from a real bronchoscopy video and used them to compare these three feature types. Figure 5 shows the results, in which the red dots are feature pixels from the first frame, the green dots are the feature pixels from the second frame and the yellow dashed lines shows the matchings of each method.

Table 1 shows the results of this comparison.

**Figure 6:**
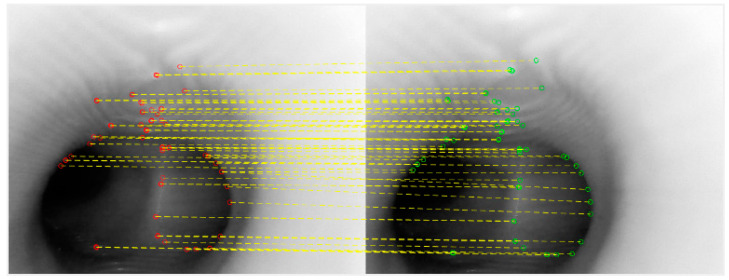
KAZE feature matching between consecutive frames.

From the results shown above, in terms of the number of correct matches, SURF would appear not to be a good choice. Examining the results of MSER and KAZE side-by-side, it would appear that a high percentage of feature points for MSER are concentrated in the center of the tunnel. Although this is not incorrect, the center of the tunnel would not result in good, representative features, as almost all frames would have the tunnel in the middle. KAZE appears to exhibit more correct feature points that are away from the center of the tunnel, and thus for this part of the study—the position tracking phase—it would appear that KAZE is the best feature descriptor. In order to verify this, a second set of consecutive frames were extracted, and the KAZE method was used. The result is quite satisfactory, as shown below in Figure 6.

For this phase, two consecutive images from the real bronchoscopy video, B, were extracted: B(t) and B(t-1). First, the KAZE feature points from the images were extracted, then matching was performed to find the approximate translation and rotation of the camera.

At the same time, the optical flow algorithm searched for neighboring sub-blocks/pixels with the highest cross-correlation; e.g., for G, a sub-block in the current frame, and H, a corresponding sub-block in the next frame, their cross-correlation can be calculated as follows:

(2) $$\mathrm{CC}\left(G,H\right)=\;\frac{\Sigma_{i=1}^{M}\Sigma_{j=1}^{N}\left(g_{\mathrm{ij}}-\bar{g}\right)\left(h_{\mathrm{ij}}-\bar{h}\right)}{\sigma_{G}\sigma_{H}}$$

where M and N are the height and width of each sub-block, g_ij_ and h_ij_ are the respective pixels values at position ij, $\bar{g}$ and $\bar{h}$ are the respective averages of pixel values of the sub-blocks, and σ_G_ and σ_H_ are the respective standard deviations of the pixel values of the sub-blocks.

**Figure 7:**
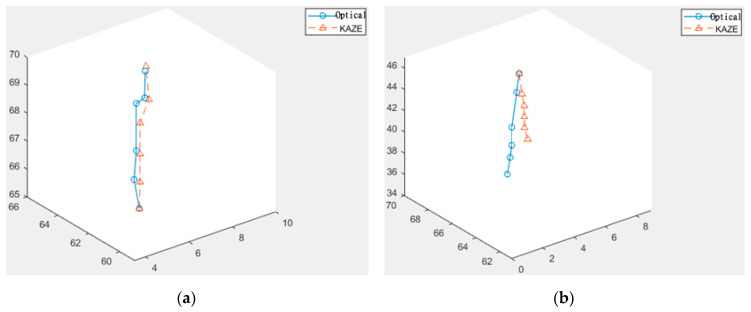
Paths calculated using KAZE and optical flow for (**a**) stable and slow movements and (**b**) with a sudden movement in the middle.

The investigation then continued with the comparison between KAZE feature-based matching and the slower optical flow methods if no verification or correction were applied. We took two short sequences from the bronchoscopy video, in which the movements of the first sequence were relatively slow and stable, and the second sequence contained a sudden movement followed by stable and slow movements. The results are shown below in Figure 7, where the blue lines are the downward paths calculated by the optical flow and the orange dashed lines are the downward paths calculated by the KAZE feature-based matching method.

After comparing the paths generated by the two different methods against the originally planned path, we find that, when the movements are slow and stable, the paths of both methods very closely approximate the originally planned path. However, when a sudden movement is introduced, one of the nodes of the KAZE path deviates from the originally planned path and would not be easily recoverable. Because KAZE is faster but appears to be more susceptible to sudden movements than optical flow, this investigation decided to design an ANFIS-based fuzzy system to decide, from node to node, whether to use the vector calculated by KAZE or wait for the optical flow results based on the suddenness of the change of motion.

**Figure 8:**
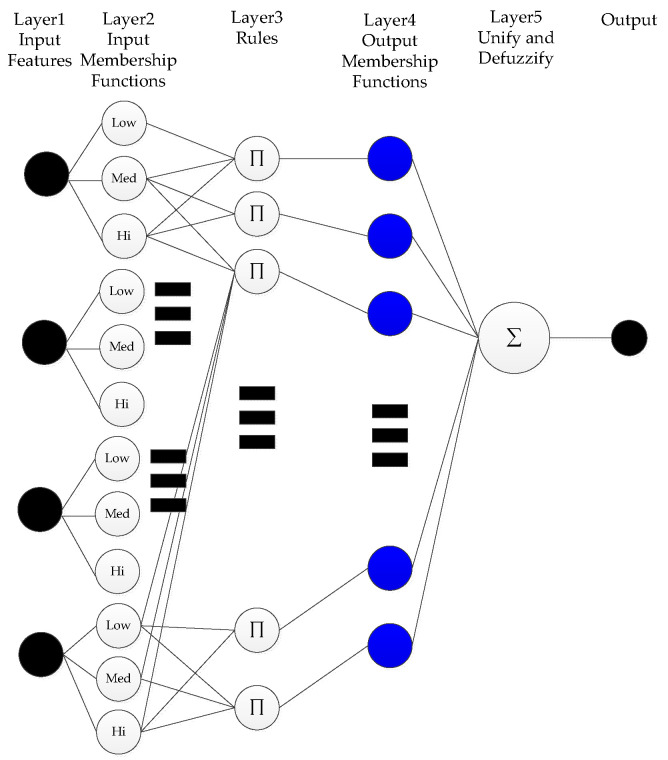
The basic architecture of the ANFIS fuzzy model.

ANFIS is an approach that integrates a fuzzy decision system with the adaptability of a neural network [22,23]. The general consensus in the published literature is that results from ANFIS are generally better than the standard fuzzy models in most cases [24,25]. The basic ANFIS architecture with fuzzy membership models for two inputs is shown below in Figure 8; for simplification purposes, not all nodes and connections are drawn. The structure is basically composed of five layers: the first layer is the input features, the second layer is the input fuzzy membership functions, the third layer is simple fuzzy if-then rules, the fourth layer contains the output fuzzy membership functions, and the last layer is simply used to unify and defuzzify the results before the output.

The results from KAZE feature matching and the optical flow modules are passed to an ANFIS-based arbitration module which, based upon the suddenness of the change of motion, decides whether the results from the optical flow module or the KAZE-based module are more accurate in estimating the change of camera position; this is a similar approach to the arbitrator in [26]. The resultant displacement—i.e., translation and rotation—is stored as an output as ΔQ and used to find the resultant Q, which is the current position. For verification and minor adjustments, ANFIS-based image matching is performed using the real bronchoscopy image against the images generated using the virtual 3D bronchial model and the displacement values.

### 2.3. Position Verification

In order to verify that the updated position computed using ΔQ matches the next frame of the bronchoscopy sequence, a similarity measure is used. The shape context determination [27] method is used to determine the similarity between two images.

**Figure 9:**
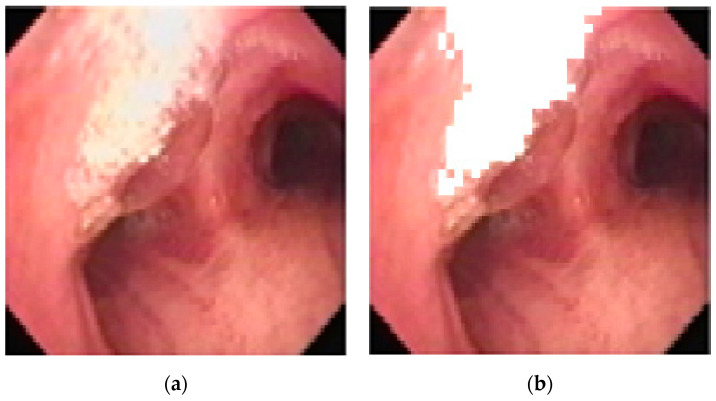
Removing areas of high reflectance from matching: (**a**) original (**b**) removed.

However, areas of high reflectance can cause error during the matching, and so they are removed from consideration prior to matching, as shown below in Figure 9.

**Figure 10:**
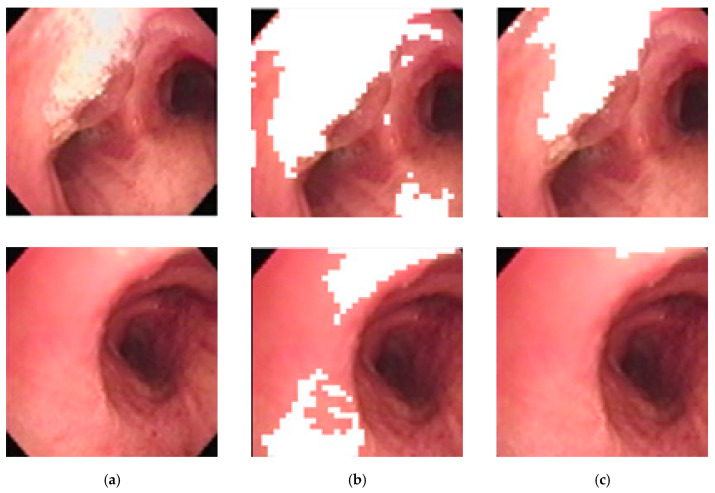
Areas of high reflectance: (**a**) original image, (**b**) threshold-based correction, (**c**) ANFIS-based correction.

The image subblocks, each of 40 × 40 pixels in size, with high reflectance are determined using an ANFIS-system using the mean saturation and mean value of an image subblock as the input, and using the COG (center-of-gravity) for defuzzification. Figure 10 below shows the differences between using a threshold vs. ANFIS-based results in determining areas of high reflectance on two random images. It is obvious that ANFIS is more accurate at determining areas of high reflectance and thus preserves more pixels for consideration.

Once the reflective-glare blocks of images are determined and removed from consideration, the image matching can begin. One problem in image matching is that the intensity distribution of some subblocks is too uniform, thus contributing nothing to image matching while taking up precious computational resources. In order to avoid wasting computing time on this type of sub-block, a simple operation is performed to determine the minimum number of non-uniform subblocks for matching. A non-uniform subblock has a high distribution of intensity values and is not near the edge between uniform subblocks and non-uniform subblocks. The regular definition of the measure of standard deviation of the intensities of any subblock is

(3) $$\mathrm{SD}=\sqrt{\frac{1}{N}\overset{N}{\underset{i=1}{\sum }}{\left(x_{i}-u\right)}^{2}}$$

where *N* is the total number of pixels in the subblock, $x_{i}$ is the intensity value of pixel *i* in the subblock and *u* is the average intensity values of all the pixels in this subblock. However, this requires square root calculation, which slows down the overall performance. Thus, instead, the $I_{\left(m,n\right)}^{\left(k\right)}$ is calculated, where *k* means the *k*th frame of the real bronchoscopy video sequence, and (*m*,*n*) is the position of the subblock within this frame, so *I*^(k)^ would be calculated for the entire frame. The definition of $I_{\left(m,n\right)}^{\left(k\right)}$ is

(4) $$I_{\left(m,n\right)}^{\left(k\right)}=\sum_{i=1}^{N}\left(x_{i}^{2}-2x_{i}u+u^{2}\right)$$

**Figure 11:**
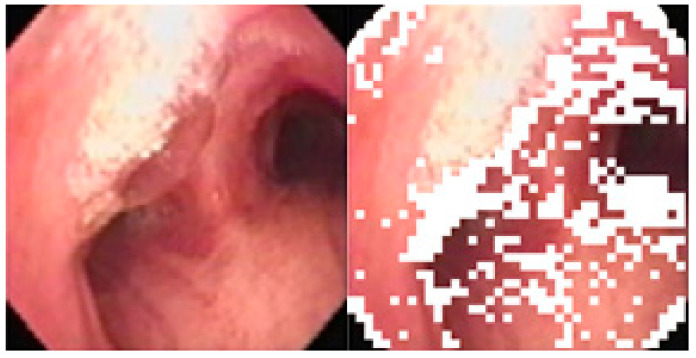
Original image and Non-uniform subblocks (marked in white).

In order to determine which subblocks are most important, a single-variable ANFIS system is built using $I_{\left(m,n\right)}^{\left(k\right)}$ as its input to produce the minimum number of non-uniform subblocks, as shown in Figure 11.

**Figure 12:**
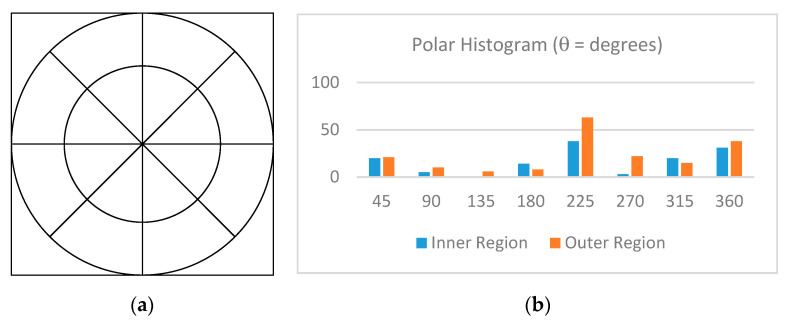
(**a**) Sixteen polar histogram regions and (**b**) histogram of non-uniform subblocks by region.

Once the non-uniform subblocks are found, image matching is only performed using the non-uniform subblocks from the actual bronchoscopy image against those from the virtual bronchoscope images. First, the polar coordinates of each is calculated using the following:

x = rcosθ, y = rsinθ, so r^2^ = x^2^ + y^2^, and θ = tan^−1^(y/x), (5)

then, the subblock is evenly divided into 16 polar regions, as shown in Figure 12a. Then, the number of important subblocks in each region is summed into a histogram as shown in Figure 12b.

Once the two polar histograms of the two images (*i* and *j*; i.e., real and virtual) to be compared are computed, Equation (6) is used to calculate their similarity measure *C_ij_*, where *h* is the number in the *k*th bin of the polar histogram, *k* is the index of sub-regions in the polar histogram and *K* is the total number of sub-regions (in this case, 16).

(6) $$C_{ij}=\frac{1}{2}\overset{K}{\underset{k=1}{\sum }}\frac{{\left[h_{i}\left(k\right)-h_{j}\left(k\right)\right]}^{2}}{h_{i}\left(k\right)+h_{j}\left(k\right)}$$

An ANFIS system is used using the *C_ij_* of the virtual frames as its input, and the de-fuzzified output determines if the virtual image is sufficiently similar to the real image. If the virtual frame is found to be of sufficient similarity to the actual image, then the location of the endoscope image is found in the virtual bronchial model. If not, then an alternative ΔQ will be used to search for a new virtual bronchoscopy image and the matching process is repeated for the new virtual bronchoscopy image. The line search method using the calculated *C_ij_* will be used to find a ΔQ that can generate a better match.

**Figure 13:**
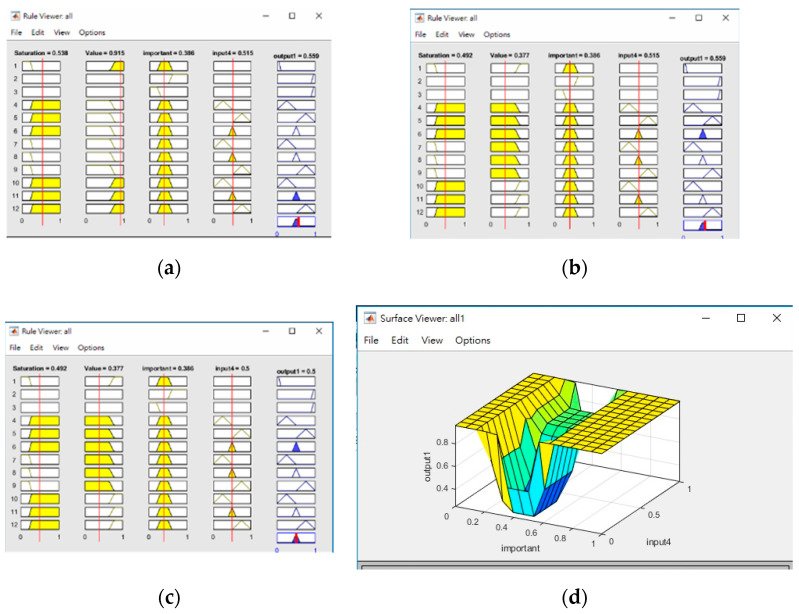
Examples of membership rules for determining (**a**) blocks with high reflectance, (**b**) important subblocks and (**c**) similarities in the shape context; (**d**) 3D visualization of the relationship between the importance of blocks vs. similarities in the shape context.

The ANFIS systems mentioned above use triangular fuzzy memberships, and Figure 13 shows sample fuzzy rules for the determination of blocks with high reflectance, the importance of blocks and the similarity of their shape context. Input 4 in the fuzzy rules represents the memberships of the shape context value, and output 1 in each figure is simply the degree to which the result would be considered as highly reflective, important or similar, respectively. The relationship between the importance of blocks vs. similarity of shape context is also shown in the 3D plot. 

### 2.4. Preliminary Study of a Hidden Markov Model-Based Path Planner

As a part of this study, a preliminary study for a path planner to aid surgeons before actual surgery was also investigated. A Hidden Markov Model (HMM) model [28,29] was used for the investigation. HMM is a stochastic method that is used to model time and series data. The HMM model can be defined using Equation (7):

μ = (S, O, A, B, π)(7)

where S = {S_1_, S_2_, S_3_, …, S_n_} represents the n hidden states set of the model; O = {O_1_, O_2_, O_3_, …, O_m_} represents the m observation set of the model; A is the transition matrix containing the possible transition from any state i to any state j with a certain probability, A = {a_ij_}; B is the emission matrix containing the emission from any state I to any observation k with a certain probability, B = {b_ik_}; and π is the initial state matrix, where π_i_ represents the probability that S_i_ is the initial starting state. The states are the numbered sequences that represent the paths that may be taken, and the other values are obtained after training the model. The HMM model requires a sufficient amount of learning/training before it can generate the desired output given an input.

**Figure 14:**
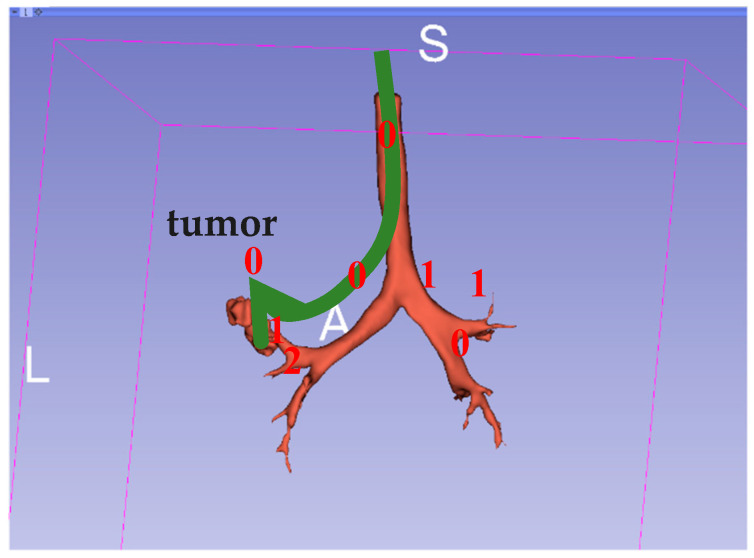
Example of a 0-0-0 path to find a bronchial tumor.

Because the bronchus has many side branches, each fork is set as a node and all branches are numbered; thus, a path from the current position of the bronchoscope to the destination is simply a sequence of numbers. Once an HMM model is built, it should be able to do the following: (1) from its current state, infer the most likely state sequence that would produce the desired output sequence; (2) infer the most likely next state, and therefore predict the output; and (3) calculate the probability that a given output sequence originates from the system model. The model can be constructed by learning from given sequences of numbers., which can be obtained from numbers encoded from previous bronchoscopy surgeries. Thus, given the source and desired destination, a trained HMM model should yield the most likely path. The Verteri-Forward algorithm was used to evaluate the probability of a specific output sequence in our model. The model was built with three states, with three output characters representing the path fork number, and the training was assumed to be converged when the average likelihood change was below 0.01. An illustration of this is shown below in Figure 14.

## 3. Results

Three sets of CT Dicom bronchial images of patients, with ages between 52 and 63, an average of around 330 slices per set, a size of 1 mm per slice per patient and a resolution of 512 × 512, were obtained with patients’ permission, and a 3D Slicer [30] was used to construct 3D bronchial models. Video sequences of actual bronchoscopy surgery for the same patients were obtained. They were taken with an Olympus BF-F260 slim broncho-videoscope [31], and OpenCV [32] and Matlab [33] were used as the software development platforms. The first experiment was set up to test whether the proposed method could locate the best-matching virtual bronchoscopy image for the given real bronchoscopy image.

### 3.1. Image Matching

**Figure 15:**
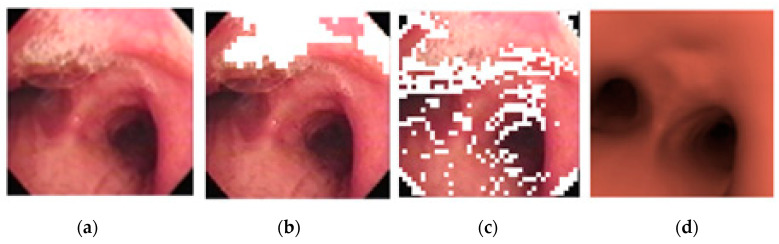
(**a**) Original bronchoscopy image; (**b**) detection of reflection-glare sub-blocks; (**c**) detection of non-uniform sub-blocks; (**d**) matched virtual bronchoscopy image.

A single bronchoscopy video sequence and its matching 3D bronchial model were used. Random images were taken, which were selected from the video sequence, and the best-matching virtual bronchoscopy image were located using the proposed method. The results were very promising; Figure 15 shows an example of the results.

### 3.2. Comparison

**Figure 16:**
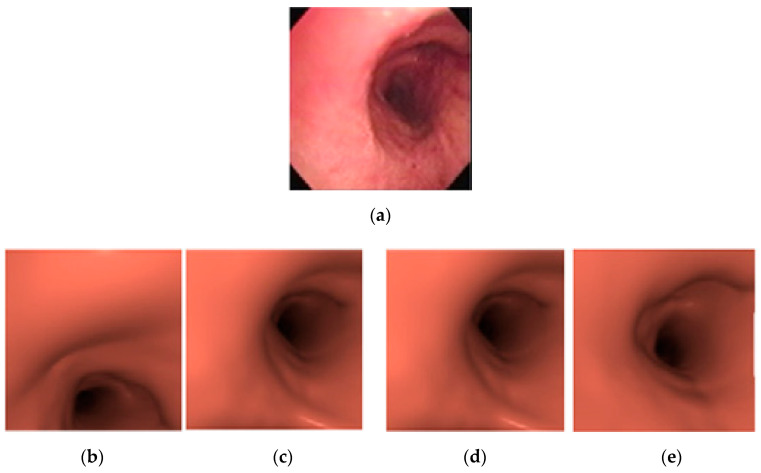
Example of matching results from different methods: (**a**) original image, (**b**) minimum square error (MSE), (**c**) modified minimum square error (MoMSE), (**d**) Improved MoMSE, (**e**) the proposed method.

The second experimental setup was used to compare the proposed method with the results using previously published methods: MSE, MoMSE and Improved MoMSE. Using the same hardware setup, the shape context similarities of the resultant virtual bronchoscopy image found using each method were measured as well as recording the execution time used to search per frame. Examples of the image results of different methods are shown in Figure 16.

**Figure 17:**
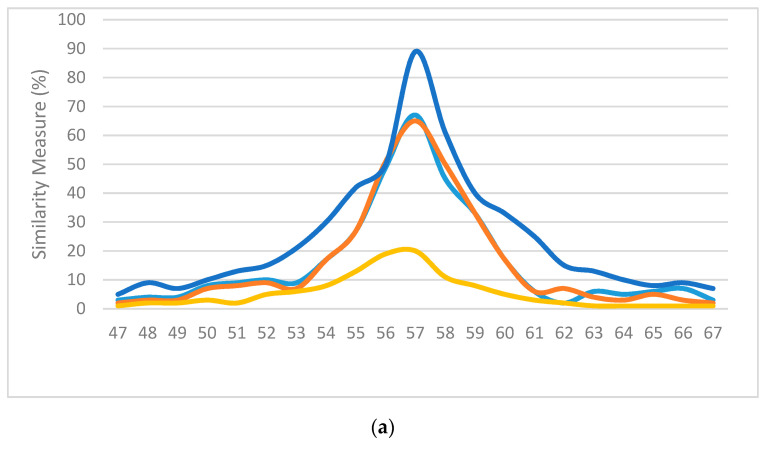

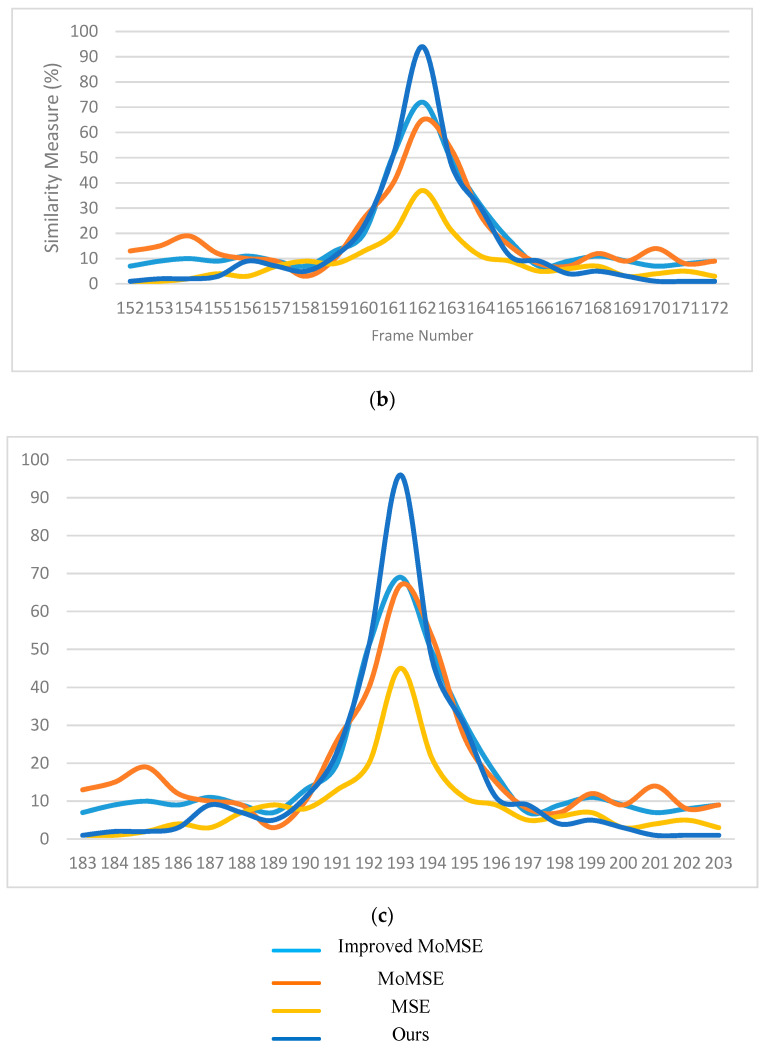
Normalized similarity measures (higher is better) of (**a**) the 57th frame of sequence #1, (**b**) the 162nd frame of sequence #2 and (**c**) the 193rd frame from sequence #3.

For subjective evaluation, the similarity measurements of various frames using different methods was compared; the higher the value, the better the match. Figure 17 displays the similarity measures of the above methods used on three random frames from three separate sequences. The blue lines represent the results of the proposed method. It can be observed from the illustrations in Figure 17 that our method had the highest probability of finding the correct location of the bronchoscope and displaying it on the virtual 3D model of the trachea compared to the other methods used in the comparison.

### 3.3. Path Navigation

**Figure 18:**
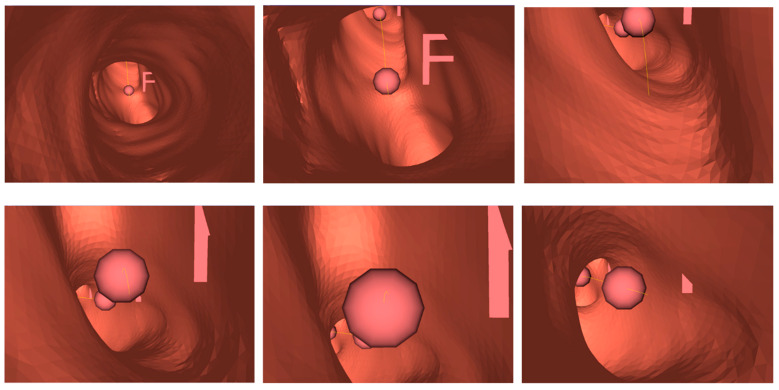
Example of a part of the path suggested by the Hidden Markov Model (HMM).

The HMM-model path model was trained using only a few bronchoscopy sequences; thus, it may not be suitable for use for all bronchoscopy contexts for different patients. This experiment was simply performed to investigate the use of a single 3D model in conjunction with a trained HMM path finder trained on this model to determine whether the bronchus junctions in the model could be correctly located and whether the path generated from the HMM model could be correctly displayed when traversing the space between the junctions. Thus, for this study, an HMM model with three hidden states was constructed and trained using only the data from three video sequences. Figure 18 shows an example of a short sequence, using balls as indicators of the existence of junctions and a thin yellow line to link the junction balls as the path suggested by the HMM model. In this experiment, the path finally led to the location of the virtual tumor.

## 4. Conclusions

In this paper, a method for bronchoscope localization using 3D virtual models and image matching was presented. Parallel processing was used to generate KAZE feature matching and optical flow vectors to approximate the camera’s motion; then, an ANFIS-based arbitrary method was used to decide which result best matched the motion of the camera and to generate the new position of the camera. Then, the new position was verified using similarity measurement between the actual bronchoscope images and generated virtual images. In the experiments, the proposed method was compared to other methods. From the experimental results, it can be seen that the proposed method shows the best measure of similarity to the original bronchoscopy image in the comparison results. This was due to the location of the bronchoscope being more correctly estimated in the tracking phase, meaning that the burden of correcting the path was much reduced in the verification phase. This novel tool could represent an aid to surgeons using a bronchoscope by allowing the simultaneous viewing of the output from the scope as well as its location so that the surgeon can correctly direct the path of the scope. Furthermore, the preliminary studies on the HMM model-based path planner showed that it is promising, but further research is required before its effectiveness in aiding surgeons can be reported. Other similarity measures and matching methods proposed in the literature [34,35] as well as other path planners mentioned in the literature, such as the Rapidly Exploring Random Trees (RRT) [36] and Probability Roadmap Planner (PRP) [37], may be investigated in future research. A comparison test with ORB-SLAM based methods may also be performed.

## Figures and Tables

**Table 1:** Comparison of the results of the three feature matching methods.

Method	SURF	MSER	KAZE
Total feature points	28	12	54
Correct matches	25	12	54
Erroneous matches	3	0	0
